# IoT Based Architecture for Model Predictive Control of HVAC Systems in Smart Buildings

**DOI:** 10.3390/s20030781

**Published:** 2020-01-31

**Authors:** Raffaele Carli, Graziana Cavone, Sarah Ben Othman, Mariagrazia Dotoli

**Affiliations:** 1 Department of Electrical and Information Engineering, Polytechnic of Bari, Via Orabona 4, 70125 Bari, Italy; graziana.cavone@poliba.it (G.C.); mariagrazia.dotoli@poliba.it (M.D.); 2 CRIStAL Laboratory UML 9189, Ecole-Central of Lille, 59655 Villeneuve d’Ascq, France; sara.ben-othman@centralelille.fr

**Keywords:** Internet of Things, Model Predictive Control, Heating Ventilation and Air Conditioning System, Predicted Mean Vote, smart buildings

## Abstract

The efficient management of Heating Ventilation and Air Conditioning (HVAC) systems in smart buildings is one of the main applications of the Internet of Things (IoT) paradigm. In this paper we propose an IoT based architecture for the implementation of Model Predictive Control (MPC) of HVAC systems in real environments. The considered MPC algorithm optimizes on line, in a closed-loop control fashion, both the indoor thermal comfort and the related energy consumption for a single zone environment. Thanks to the proposed IoT based architecture, the sensing, control, and actuating subsystems are all connected to the Internet, and a remote interface with the HVAC control system is guaranteed to end-users. In particular, sensors and actuators communicate with a remote database server and a control unit, which provides the control actions to be actuated in the HVAC system; users can set remotely the control mode and related set-points of the system; while comfort and environmental indices are transferred via the Internet and displayed on the end-users’ interface. The proposed IoT based control architecture is implemented and tested in a campus building at the Polytechnic of Bari (Italy) in a proof of concept perspective. The effectiveness of the proposed control algorithm is assessed in the real environment evaluating both the thermal comfort results and the energy savings with respect to a classical thermostat regulation approach.

## 1. Introduction

In recent times, the increase of energy efficiency is a pivotal goal for energy policy makers that aim at promoting a conscious, cost-effective, and sustainable energy use in management of resources and infrastructures [1], transportation and logistics [2], the production and industrial sector [3], and all activities related to human life [4]. A significant part of energy consumption concerns the energy demand of buildings that in Europe amounts to 40% of the total and is mainly due to the so-called Heating, Ventilation and Air Conditioning (HVAC) systems [5]. HVAC systems are devoted to guarantee hygrothermal comfort in building indoor environments [6] and their automated management can largely impact the virtuous behavior of its end-users. In particular, it is of paramount importance to implement novel control architectures that on the one hand allow the energy optimization of HVAC systems without neglecting the thermal comfort of building occupants and on the other hand offer to the policy makers and citizens (i.e., the end-users) an interactive tool for the monitoring and control of the HVAC system. This can be achieved by combining two main modules: (1) a control algorithm and (2) a smart physical infrastructure. The first module must be devoted to the energy and thermal comfort optimization while the second takes care of the sensing, communication, data storage, and actuation of the HVAC system on the basis of the end-users’ requests.

For the control algorithm module, many techniques can be considered that have been developed during the years for HVAC systems. However, the majority of them consist typically in the intuitive on/off controllers that cannot compensate for the high thermal inertia of many HVAC processes, or in simple PID controllers whose tuning is a complex activity and whose performance degrades if the system conditions vary [7]. Only in the last two decades the more promising Model Predictive Control (MPC) approach is taking off: this control technique allows to effectively integrate issues such as disturbance rejection, constraint satisfaction, and slow-moving dynamic control together with energy efficiency strategies into the controller formulation. Furthermore, thanks to the decreasing costs of smart devices, the large availability of distributed sensors and data analytics tools, and in general the advances of Information and Communication Technology (ICT) [8,9], the implementation of optimal control approaches for the energy efficiency and thermal comfort optimization is becoming more immediate and affordable [10]. It is then evident that MPC becomes useless if it is not associated to a proper smart physical infrastructure that allows the collection/forwarding of actual data from/to the field [11]. The Internet of Things (IoT) offers a proper solution by allowing the connection of sensors, actuators, and other objects to the Internet, and thus permitting the perception of the world, as well as the interaction with it [12,13].

In this paper, we propose an IoT based architecture for the implementation of MPC of HVAC systems in smart buildings. The system is composed by a set of smart sensors and actuators, a gateway, a database server, a control unit, and a user friendly interface or dashboard, which are all networked and connected to the Internet. The considered control algorithm optimizes on line, in a closed-loop control fashion, both the indoor thermal comfort and the related energy consumption for a single zone environment. The end users can retrieve information about comfort and environmental indices, while they are also able to remotely configure the temperature and the control mode of the system.

The remainder of this paper is organized as follows. Section 2 presents an overview of the related works on the optimal control algorithms in this field and the positions of the contributions of this paper within the reviewed state of the art. Section 3 describes the proposed IoT based sensing, control, and actuating architecture and the HVAC system model under study. Section 4 introduces the MPC based control algorithm in an energy efficiency and thermal comfort optimization context. Section 5 provides the description of the implemented IoT based control architecture and the experimental results on a real case study. Finally, Section 6 concludes this paper.

## 2. Related Works and Paper Contributions

The first applications of MPC to the HVAC system control, see, e.g., [14,15], mainly consider the minimization of the energy consumption and the limitation of the temperature inside a constrained range. Evidently, bounding and controlling only the temperature does not imply the maximal comfort of occupiers [16]. Therefore, the Predicted Mean Vote (PMV) index (standardized by ISO [17]) has been introduced to comprehensively express the thermal comfort conditions [7,18] and has been subsequently combined with the MPC technique with the aim of optimizing the indoor thermal comfort in smart building environments. However, the non-linear nature of the PMV index represents a significant challenge in the design and implementation of such control frameworks. In effect, the MPC controller is required to solve, at each sampling time, a non-linear optimization problem that evidently requires a large amount of computational resources and can lead to long computation times [16]. Furthermore, the non linearity of the PMV limits the applicability and scalability of the control problem formulation. The approaches proposed in the related literature to mitigate the recalled limitations typically consist in defining approximated and linearized versions of the PMV [19] that can be easily computed and efficiently performed even on simple hardware. The contributions that propose the use of a linearized PMV for the MPC technique can be classified in two main groups: (1) the PMV thermal comfort index is included in the constraints set of the optimization problem; (2) the PMV index is a term of the objective function and its values are subject to soft constraints.

For the first type of approach, Xu et al. [20] propose a building operational optimization method based on a piecewise linearization approximation of the PMV to be included in the constraints set, which allows the reduction of the computational effort with respect to the standard formulation. Similarly, Alamin et al. [21] propose an economic MPC approach to control the thermal comfort of a bioclimatic building room using a first order linear time-invariant system for modelling the relation between the PMV index and the speed of fan coil units. In [16] the authors formulate the MPC problem as a parametric quadratic program and include a linearized version of the PMV index in the constraint set of the problem. Furthermore, with the aim of simplifying the resolution of the optimization problem, authors reformulate the MPC optimization problem in an explicit form.

For the second type of approach, the PMV index is explicitly incorporated into the MPC cost function and soft constraints are considered to limit the PMV in a certain range. For instance, Cigler et al. [22] define a receding horizon optimization method aiming at keeping the temperature of the supply water and the PMV index inside a given range based on a linear approximation of the PMV. Corbin et al. [23] instead implement an MPC procedure where the objective of the optimization is the minimization of the energy consumption and of a penalty term calculated from comfort violation. The optimal building control strategies are determined by a modified particle swarm optimizer in order to achieve acceptable computational performances. In addition, using genetic algorithms, the approach proposed by Ascione et al. [24] enables the computation of a set of multi-objective control strategies, among which occupants can choose a trade-off solution compliant both to comfort and energy saving needs.

It has to be highlighted that all the cited contributions consider an implicit MPC formulation where the control law is defined by solving the optimization problem in real-time, however in most of the cited works [16,20,22,23,24], all the proposed algorithms are tested in a simulation environment only, thus without providing information on the actual control architecture implementation. This paper overcomes this important limitation.

We underline that this work extends our baseline article [25], where we present a preliminary version of our MPC algorithm aiming at optimizing the thermal comfort and energy efficiency of indoor environments. In [25] we consider the optimization of a non-linear thermal comfort index together with a fixed cost energy consumption function, subject to strict constraints on comfort requirements. This results in a non-linear optimization problem that on the one hand allows the precise representation of the thermal comfort, but on the other hand prevents the applicability of the technique in case of partial knowledge of the thermal parameters of the considered environment and in case of physical limits of the actuation system. In this paper, we consider an implicit and tractable MPC algorithm for HVAC control to minimize the energy consumption while maintaining the PMV into a desired range and providing a smooth control action. Differently from the above, we define a general IoT based control architecture that allows the application of the MPC control algorithm to the actual system and the inclusion of the end user in the control loop. The contributions of this work can be thus summarized as follows.

- We propose a dynamic energy and thermal comfort controller based on the MPC technique, which selects the most efficient configuration of HVAC system that satisfies the users’ needs. More in detail, we formulate an optimization problem of HVAC control based on a simplified thermal model of a single zone environment and on a linearized version of the non-linear Fanger’s PMV index representative of the users thermal comfort. Differently from the state of the art, our energy and thermal comfort control approach both considers the constraints on comfort as a penalty term in the objective function and takes into account the eventual physical limitations of the real system (e.g., physical limitations of the actuators or of the HVAC system), thus ensuring the feasibility of both the optimization problem and control action implementation. This results in a quadratic optimization problem formulation that aims at simultaneously maximizing the users’ thermal comfort, the energy efficiency of the HVAC system, and the smoothness of the control action, while minimizing the comfort limits violations.
- We propose a general IoT based control architecture that can be easily implemented in a real smart building environment for the HVAC system control. Contrary to the existing contributions, which consider closed systems using local networks, our IoT based approach allows the implementation of a smart control system where the sensors, actuators, and control unit are connected to the Internet and can take advantage of the functionalities of external database servers and API. Moreover, an end-user device also connected to the internet allows the interaction of the building occupants with the control system by means of a user friendly dashboard.
- We present a real IoT based control architecture test bed to evaluate the performance of the proposed approach, demonstrating the potential financial and comfort gains that can be achieved and the effectiveness of the method in the real-time control environment in a proof of concept perspective. In this work, differently from the state of the art that exclusively focuses on the control algorithm implementation or on the system architecture definition, we provide the description of the HVAC control architecture as a whole for practical use in a real environment.

## 3. IoT based Control System Architecture

### 3.1. System Architecture

**Figure 1:**
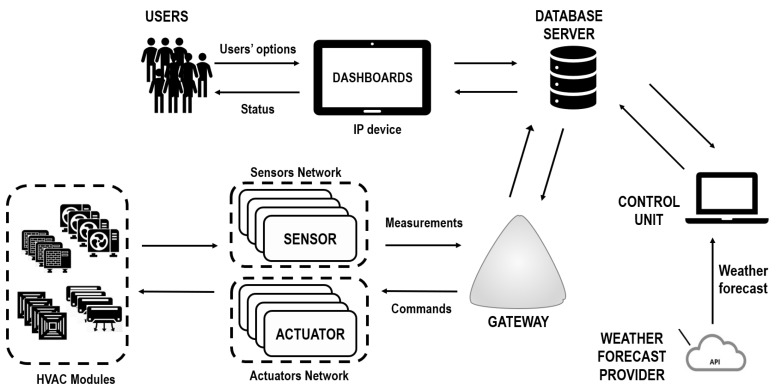
The high-level system diagram of the proposed IoT based architecture.

Figure 1 presents the overall IoT based control system architecture which allows to optimize the energy efficiency and thermal comfort of the internal environment of smart buildings. It consists of the following elements:

- a net of sensors that perceives the environmental conditions and sends measurements to a gateway;
- a set of HVAC modules;
- a net of actuators that control the HVAC modules and communicate with the gateway;
- a gateway that connects the nets of sensors and actuators to the Internet;
- an external Application programming interface (API) that provides forecasts of the weather conditions;
- an external database server that collects/forwards data from/to the field and from/to the control unit;
- a control unit that communicates with the database server and where the MPC algorithm is executed;
- an IP device that acts as end-user interface that is connected to the database server and hosts a dashboard dedicated to monitoring the state of the environment and setting the control system mode.

The mode of operation and flow of information of the proposed architecture are as follows. The environmental indoor conditions (i.e., temperature, CO${}_{2}$ level, number of occupants, etc.) and the energy consumption are measured by means of the net of sensors. Then, the measurements are periodically sent to the gateway, which communicates with the database server, and then to the control unit where the MPC algorithm is deployed. This algorithm provides to the HVAC systems the control actions that ensure the best compromise in terms of energy consumption and comfort for the given comfort constraints during a particular time horizon. These control actions are sent via the gateway to the devices that actuate the HVAC modules. The HVAC modules modify the room temperature according to the decisions taken by the MPC algorithm. Moreover, the database server stores the measurements of temperature and energy consumption. These measurements are displayed at the end user by means of the dashboard on an IP device, which is connected to the database server. The dashboard allows users to interact with the control unit and select the desired temperature and the desired control mode (i.e., manual or automatic).

### 3.2. The Thermal Model of a Building Single Zone Environment

In this paper we consider a discrete-time model where time slots of equal duration are indexed by k∈N. The evolution of the internal temperature of the considered indoor environment (i.e., a building zone or a room) equipped with a HVAC system can be described with a linear discrete-time differential equation [26] that takes into account the complexity of the system (i.e., the presence of various and heterogeneous elements) and the influence of the thermal condition of the external climate and contiguous rooms. In particular, assuming that the initial instant is zero (i.e., initial conditions correspond to k=0), we have:

(1) $$T\left(k\right)=e^{-\frac{\Delta h}{\tau }}T\left(k-1\right)+\left(1-e^{-\frac{\Delta h}{\tau }}\right)\left(T_{e}\left(k-1\right)+Q_{f}u\left(k\right)\right)=f_{k}\left(T\left(k-1\right),u\left(k\right)\right),\;k\in N\setminus \left\{0\right\}$$

where Δh is the sampling time, τ is the constant time of the first order dynamic of the building zone temperature, T(k−1) is the temperature of the environment measured at time k−1, $T_{e}\left(k\right)$ is the external measured temperature at time *k*, $Q_{f}$ is the total heating/cooling gain due to a HVAC system in the considered environment ($Q_{f}$>0 if the HVAC system is in heating mode and $Q_{f}$<0 if the HVAC system is in cooling mode), and u(k) is the control signal provided to the HVAC system (e.g., fan speed of a fan coiler unit).

It has to be highlighted that, since this work aims at offering a baseline algorithm that can be scaled and applied to a variety of buildings, we consider the use of a linear thermal model so as to allow an easy re-use of the method in its current form or eventually after a proper customization (with minimum extra implementation work) to a different environment. Moreover, the linearity of the thermal model also allows to speed up the resolution of the MPC algorithm. We can also consider that the heating/cooling of the environment requires an energy consumption necessary for keeping an adequate thermal comfort that is proportional to the exchanged heat:

(2) $$E\left(k\right)=\eta |Q_{f}|u\left(k\right),\;k\in N\setminus \left\{0\right\}$$

where η is the thermal efficiency of the HVAC system.

## 4. The Control Algorithm

In this paper the control algorithm is based on the MPC technique. It includes a quadratic programming optimization problem whose resolution aims at ensuring the optimal balance between the thermal comfort and the energy consumption. The optimization problem is based on the thermal model presented in Section 3 and we consider the PMV as thermal comfort index. In the next subsections first the PMV and the basics on the MPC technique are provided, then the optimization problem is detailed.

### 4.1. Thermal Comfort Assessment by PMV

The Predicted Mean Vote, developed in the seventies by Fanger [18], is the most used thermal comfort index in the related literature. In particular, it allows to assess the global thermal comfort and to predict the mean value of the votes of a large group of people that perform similar activities, in similar clothing conditions, and in the same place, which is uniform with respect to its related physical characteristics, i.e., air temperature, air speed, air relative humidity, and mean radiant temperature. Fanger’s original formulation of the PMV index is complex and non-linear, as it relates the recalled environmental parameters with the individual metabolism and the clothing conditions. The value of the PMV index is dimensionless and ranges between seven possible comfort levels comprised in the ±3 interval. The zero value represents the optimal thermal comfort condition. Values between ±1 correspond to slightly cold/warm conditions, PMV = ±2 expresses cool/warm perception, and values above ±3 indicate unpleasantly cold/hot conditions in the building.

Hereafter we report the non-linear PMV index formulation [18], which is valid for a semi-permanent regime and internal air temperature ranging between 10 ÷ 30 °C [17]:

(3) $$PMV=(0.303e^{-0.036M}+0.028)L$$

where:

(4) $$\begin{array}{cc} L= & M-W-3.0510^{-3}\left(5733-6.99\left(M-W\right)-P_{a}\right)-0.42\left(M-W-58.15\right)+\\ \; & -1.7210^{-5}M\left(5867-P_{a}\right)-0.0014M\left(34-T\right)+\\ \; & -3.9610^{-8}f_{cl}\left({\left(T_{cl}+273.16\right)}^{4}-{\left(T_{mr}+273.16\right)}^{4}\right)-f_{cl}h_{c}\left(T_{cl}-T\right) \end{array}$$

(5) $$\begin{array}{cc} T_{cl}= & 35.7-0.028\left(M-W\right)-I_{cl}(3.9610^{-8}f_{cl}({\left(T_{cl}+273.16\right)}^{4}-{\left(T_{mr}+273.16\right)}^{4}+\\ \; & +f_{cl}h_{c}\left(T_{cl}-T\right)) \end{array}$$

(6) $$h_{c}=\left\{\begin{array}{cc} 2.38|T_{cl}{-T|}^{0.25} & \mathrm{if}\;2.38|T_{cl}{-T|}^{0.25}\geq 12.1\sqrt{V_{ar}}\\ 12.1\sqrt{\left(V_{ar}\right)} & \mathrm{if}\;2.38|T_{cl}{-T|}^{0.25}<12.1\sqrt{V_{ar}} \end{array}\right.$$

(7) $$f_{cl}=\left\{\begin{array}{cc} 1.00+1.290I_{cl} & \mathrm{if}\;I_{cl}\leq 0.078\\ 1.05+0.645I_{cl} & \mathrm{if}\;I_{cl}>0.078. \end{array}\right.$$

The variables and parameters used in Equations (3)–(7) are defined in Table 1: most quantities can be measured by sensors (e.g., air temperature, velocity, and humidity), whilst the remaining ones can be estimated based on the type of occupancy type and level (e.g., occupants activity and clothing).

With the aim of defining a computationally tractable optimization problem for the MPC based HVAC control, we here provide a linearized version of the PMV index that can be applied to efficiently compute the thermal comfort in indoor environments. For the formulation of the PMV index we make some simplifying assumptions based on the definition of the comfort ranges in the ASHRAE standards [27], the NORDTEST standard protocol developed for testing the moisture buffering value of materials [28], and the ISO 7730 standard [17] as follows:

- Energy metabolism *M*—for sedentary activities in offices or housing it is equal to 70$\frac{W}{m^{2}}$,
- Thermal insulation of clothing $I_{cl}$—it is the sum of a term corresponding to work clothing (for office) equal to 0.14 $m^{2}\frac{K}{W}$ and the thermal insulation of a typical chair equal to 0.016 $m^{2}\frac{K}{W}$;
- Area coefficient of clothing $f_{cl}$—for the $I_{cl}$ value chosen above, the second of the formulas in Equation (7) must be considered, that is $f_{cl}$ = 1.05 + 0.645$I_{cl}$;
- Effective mechanical power *W*—for sedentary activities it can be assumed equal to zero;
- Average radiant temperature $T_{mr}$—with good approximation it is assumed the same as air temperature, which happens in latest generation buildings;
- Air speed $v_{ar}$—as explained in [28] the air velocity in moderate work environments should be kept in the interval 0.15 ÷ 0.25 [ms] and it can be considered constant and equal to 0.1 [ms].

We consider a linearized version of the PMV which is a function of the indoor air temperature *T* and of the absolute humidity of the air ψ$\left[g_{H_{2}O}/m^{3}\right]$. To this aim, based on the air condensation rules, we express the partial pressure of the water vapor in the air $P_{a}$ as:

(8) $$\begin{array}{c} P_{a}=6.11\frac{\varphi }{100}10^{\frac{7.5(T-273.15)}{237.7+(T-273.15)}} \end{array}$$

where we denote the relative humidity as φ∈[0,1] and the temperature *T* is expressed in degrees Kelvin. The relative humidity depends on the absolute humidity according to the rule:

(9) $$\begin{array}{c} \varphi =\frac{\psi }{\psi_{sat}}=\frac{\psi }{U_{s,max}\rho_{a}} \end{array}$$

where $\psi_{sat}$ is the absolute humidity of saturation, which can be expressed as the product of the maximal specific saturation humidity $U_{s}$$\left[g_{H_{2}O}/{\mathrm{kg}}_{\mathrm{air}}\right]$ and the air density at atmospheric temperature and pressure $\rho_{a}$$\left[{\mathrm{kg}}_{\mathrm{air}}/m^{3}\right]$. The saturation specific humidity $U_{s}$ is function of the saturation vapour pressure $P_{v,sat}$ and of the atmospheric pressure *P* as follows [29]:

(10) $$\begin{array}{c} U_{s}=\frac{A\;P_{v,sat}}{\frac{P}{100}-B\;P_{v,sat}} \end{array}$$

where constant A=622 and the constant B=0.378 (both these constants are dimensionless). The saturation vapor pressure $P_{v,sat}$[Pa] is defined by the equation proposed by Murray [30] as follows:

(11) $$\begin{array}{c} P_{v,sat}=a\;10^{\frac{b(T-273.15)}{c+(T-273.15)}} \end{array}$$

where the constant a=6.11 [Pa], while the dimensionless constant b=7.5 and the constant c=237.7 [K]. It is then possible to express the relative humidity ϕ as:

(12) $$\begin{array}{c} \varphi =\psi \frac{RT}{P}\frac{\frac{P}{100}-0.378\;6.11\;10^{\frac{7.5(T-273.15)}{237.7+(T-273.15)}}}{622\;6.11\;10^{\frac{7.5(T-273.15)}{237.7+(T-273.15)}}} \end{array}$$

where *R*$\left[J\;{\mathrm{kg}}^{-1}\;K^{-1}\right]$ is the universal gas constant for molar mass of dry air and P=101,300$P_{a}$ [Pa] is the atmospheric pressure.

Substituting Equation (8) and Equation (12) in Equation (4) it is possible to obtain PMV as a non-linear function of ψ, *T*, and $T_{cl}$. We then linearize the clothing temperature function of Equation (5) considering that in comfort situation the air temperature *T* varies in the interval 20 ÷ 22 °C. In particular, we consider a linear approximation of the clothing temperature for the air temperature variation interval 17 ÷ 25 °C. Finally, the non-linear PMV index is linearized around the zero PMV work point that corresponds to $T_{L}$ = 21.4 °C and $\psi_{L}=10$$\left[g_{H_{2}O}/m^{3}\right]$. The formulation of the linearized PMV is then equal to:

(13) $$PMV^{\prime }=h_{1}\left(T-T_{L}\right)+h_{2}\left(\psi -\psi_{L}\right)$$

where we mark the linearized PMV symbol with an apex in order to differentiate it from the exact PMV symbol as defined in Equations (1)–(7) and we introduce the coefficients $h_{1}$ = 0.20 and $h_{2}$ = 0.03 that result from the linearization procedure.

### 4.2. Model Predictive Control

During the last two decades, a great effort has been devoted by the scientific community to implement MPC based control strategies for HVAC systems. The main reason for such a large research investment lies in the possibility of performing both feedback control and performance optimization [31] with a single control law. In general, the MPC technique requires the definition of three main elements: (1) a dynamical model of the controlled system, which must be used to predict the evolution of the system in response to the application of the control actions, (2) a prediction and a control horizon over which the dynamics of the system behavior and of the control actions have to be computed, (3) a time step, in which an optimization problem is solved over the chosen control rolling horizon. At each time step, information on the system behavior is gathered and used to update the dynamics of the system. Subsequently, the optimization problem is solved over the control horizon and the resulting control actions are applied to the system in a closed-loop control mode. Note that the control actions are applied only in the time step subsequent to the observation of the current system state. The procedure is then iteratively executed by making the horizon roll into the future until the end of the time interval of interest [32].

**Figure 2:**
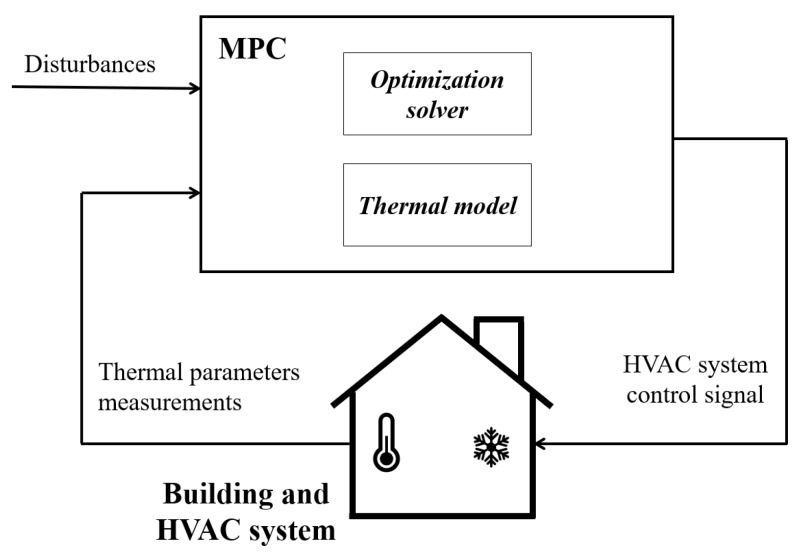
The proposed framework of MPC integrating the thermal model of the indoor environment.

Figure 2 shows the MPC framework proposed in this paper: the MPC integrates both the control oriented building zone and HVAC system models (described in the previous section), taking into account the mutual interaction between the building zone thermal behaviour and the HVAC energy components. The MPC law is defined in accordance with an output-feedback formulation. The on-line optimization problem aims at determining the controlling variables (e.g., HVAC actuators manipulated inputs), whilst the measured responses coincide with the main thermal parameters (e.g., indoor temperature, humidity, etc.) monitored by the available sensors deployed in the indoor environment. The estimation of all the variables influencing the thermal comfort which are not monitored by sensors as well as the presence of disturbances affect the accuracy of the model response.

### 4.3. The Optimization Problem

In this paper the MPC scheme solves over a predefined time horizon a quadratic optimization problem which is based on the PMV formulation and the thermal model provided in the previous sections. In particular, at each time slot *k*, the system behavior is observed and information is collected and used to update the dynamical thermal model of the system. Then, the optimization problem is solved over a prediction horizon including *J* time slots and the results are applied to the system for one time slot in a closed-loop control fashion (note that we consider the same length for both the prediction and control horizon). The procedure is iterated until the automatic control scheme is active.

In particular, we consider the resolution of the following optimization problem at each time slot *k*:

(14) $$\begin{array}{c} \min\;w_{1}\sum_{j=k+1}^{k+J}\mu \left(j\right){\left[P\left(j\right)-P_{ref}\left(j\right)\right]}^{2}+w_{2}\sum_{j=k+1}^{k+J}\lambda \left(j\right){\left[E\left(j\right)\right]}^{2}+w_{3}\sum_{j=k+1}^{k+J}{\left[u\left(j\right)-u\left(j-1\right)\right]}^{2} \end{array}$$

s.t.

$$\begin{array}{ccc} P(j) & =h_{1}\left(T\left(j\right)-T_{L}\right)+h_{2}\left(\psi \left(j\right)-\psi_{L}\right),\; & j\in [k+1,k+J]\\ \psi (j) & =\psi (k),\; & j\in [k+1,k+J]\\ T(j) & =f_{j}\left(T\left(j-1\right),u\left(k\right)\right),\; & j\in [k+1,k+J]\\ E(j) & =\eta |Q_{f}|u\left(j\right),\; & j\in [k+1,k+J]\\ P_{min} & \left(j\right)\leq P\left(j\right)\leq P_{max}\left(j\right),\; & j\in [k+1,k+J]. \end{array}$$

The objective function in Equation (14) is composed by three terms, weighted by coefficients $w_{1}$, $w_{2}$, and $w_{3}$, respectively. The first one is the evolution, over the *J* time slots of the prediction horizon, of the quadratic error of the PMV index with respect to its reference signal $P_{ref}$ (i.e., the most comfortable value of PMV). Note that μ(j) is a time-varying parameter that gets value 0 (or 1) if time slot *j* belongs to the time window when the comfort conditions must be (not be) guaranteed. Note that the end-users can set the occupancy time window for the considered controlled zone, thus it is assumed that in the corresponding time window people are present in the controlled zone. The second term in the objective function of Equation (14) is the evolution, over the *J* time slots of the prediction horizon, of the quadratic deviation of energy consumption with respect to a reference signal equal to 0 (i.e., no energy consumption). Note that λ(j) is a time-varying coefficient that models the prioritization of energy savings over the time horizon (for instance when the exploitation of the building local generation has to be maximized). We further remark that λ(j) can alternatively be used to model the energy pricing in case the on-line optimization is intended to pursue building operational cost savings (cost efficient buildings) rather than energy savings (energy efficient buildings such as near zero energy buildings). The third term in the objective function of Equation (14) is the evolution, over the *J* time slots of the prediction horizon, of the quadratic variation of the control signal between two consecutive steps and aims at avoiding undesired fluctuations that will possibly damage the actuators. As for the constraints in Equation (14), the first one defines the PMV index in accordance with Equation (13). The second one defines a constant projection, over the *J* time slots of the prediction horizon, of the absolute humidity of the air measured by sensors at time slot *k*. It has to be noticed that in a small/medium zone the absolute humidity is rather homogeneous and well mixed; consequently, the absolute humidity can be considered almost constant. The third and fourth one is related to the thermal and energy model previously described in Equation (1) and Equation (2), respectively. The fifth one ensures that in the whole time horizon the PMV index lies in the comfort range defined by $P_{min}$ and $P_{max}$. Note that $P_{min}$ and $P_{max}$ are time varying bounds defined by the particular class of ISO 7730 norm when occupants are present.

It is worth noting that the proposed optimization problem includes both a tractable time varying formulation of the indoor environment thermal dynamics and a linearized version of the PMV index. In this way, we limit the effort necessary for the computation of the control action (details on computation times are provided in the following section). Furthermore, the third term of the objective functions allows smoothing the control input to avoid undesired rapid oscillations of the control signal.

Furthermore, we remark that the stability of the above defined optimal control system is unquestioned, since the addressed system (i.e., thermodynamical process in building single zone) is generally affected by long response times. Conversely, the feasibility of the optimization problem at time slot *k* defined by Equation (11) is a concern. To this aim, hereafter, we provide a more flexible formulation of Equation (11) that can be used to ensure the feasibility of the problem in particularly noisy environments where disturbances (e.g., high variability of occupants, adverse weather conditions, substantial variations of the absolute humidity) can drive the plant into a state for which the thermal comfort constraints cannot be strictly satisfied and a new control input cannot be computed without any relaxation of the constraints in Equation (14).

(15) $$\begin{array}{cc} \min\;w_{1}\sum_{j=k+1}^{k+J}\mu \left(j\right){\left[P\left(j\right)-P_{ref}\left(j\right)\right]}^{2}+ & w_{2}\sum_{j=k+1}^{k+J}\lambda \left(j\right){\left[E\left(j\right)\right]}^{2}+\\ & w_{3}\sum_{j=k+1}^{k+J}{\left[u\left(j\right)-u\left(j-1\right)\right]}^{2}+w_{4}\sum_{j=k+1}^{k+J}\left[\bar{p}{\left(j\right)}^{2}+\underline{p}{\left(j\right)}^{2}\right] \end{array}$$

s.t.

$$\begin{array}{ccc} P(j) & =h_{1}\left(T\left(j\right)-T_{L}\right)+h_{2}\left(\psi \left(j\right)-\psi_{L}\right),\; & j\in [k+1,k+J]\\ \psi (j) & =\psi (k),\; & j\in [k+1,k+J]\\ T(j) & =f_{j}\left(T\left(j-1\right),u\left(k\right)\right),\; & j\in [k+1,k+J]\\ E(j) & =\eta |Q_{f}|u\left(j\right),\; & j\in [k+1,k+J]\\ \bar{p}\left(j\right) & \geq 0,\; & j\in [k+1,k+J]\\ \underline{p}\left(j\right) & \geq 0,\; & j\in [k+1,k+J]\\ \bar{p}\left(j\right) & \geq P\left(j\right)-P_{max}\left(j\right),\; & j\in [k+1,k+J]\\ \underline{p}\left(j\right) & \geq P_{min}\left(j\right)-P\left(j\right),\; & j\in [k+1,k+J]. \end{array}$$

Comparing Equation (14) with Equation (15), we remark that in the latter formulation we relax the strict constraint $P_{min}\left(j\right)\leq P\left(j\right)\leq P_{max}\left(j\right)$ of Equation (14) by introducing the slack variables $\bar{p}\left(j\right)$ and $\underline{p}\left(j\right)$ and modifying both the objective function and the constraints set as follows:

- The slack variable $\bar{p}\left(j\right)$ takes into account the case when the PMV exceeds the occupant-defined $P_{max}$ above, whilst it is zero otherwise. Similarly, the slack variable $\underline{p}\left(j\right)$ takes into account the case when the PMV exceeds the occupant-defined $P_{min}$ below, whilst it is zero otherwise.
- The objective function of Equation (15) contains an additional term with respect to Equation (14) that is weighted by the constant $w_{4}$. This term represents a penalty to be added in case of violation of the PMV bounds. More in detail, based on the definition of the two slack variables, the fourth term of the objective function in Equation (15) is straightforwardly the sum of the squares of $\bar{p}\left(j\right)$ and $\underline{p}\left(j\right)$.
- The constraints $P_{min}\left(j\right)\leq P\left(j\right)\leq P_{max}\left(j\right),\;j\in \left[k+1,k+J\right]$ in Equation (14) are replaced by the last four constraints in Equation (15).
  The first two constraints impose the two slack variables to be non-negative; whereas the second two constraints impose the slack variables to be higher than or equal to the excess of the thermal comfort with respect to its limits.

## 5. Case Study

The proposed IoT based MPC architecture is applied to a real office building of the University campus of the Polytechnic of Bari (southern Italy) and belonging to the Department of Electrical and Information Engineering. The building was built in the seventies and it has five floors devoted both to administrative and research activities. The thermal regulation of the whole building depends on a central HVAC system that provides hot and cold water (during the cold and hot season, respectively) to the Fan Coil Units (FCUs) mounted in each room. The thermal exchange of the FCUs with the rooms can be individually adjusted and regulated by means of a variable speed fan that inflows hot/cold air in the environment; the air is warmed/refreshed by means of a heat exchanger. For the scopes of this research work, in one of the laboratories in the building, a network of smart sensors and actuators is installed; all the monitored and controlled data are collected in a database server and are available to the end-users via suitable dashboards for management and analysis purposes. The laboratory then operates both as a research center and demo site with the final goal of defining and testing the IoT based control architecture for the thermal comfort and energy efficiency optimization.

### 5.1. System Set-Up and Architecture

**Figure 3:**
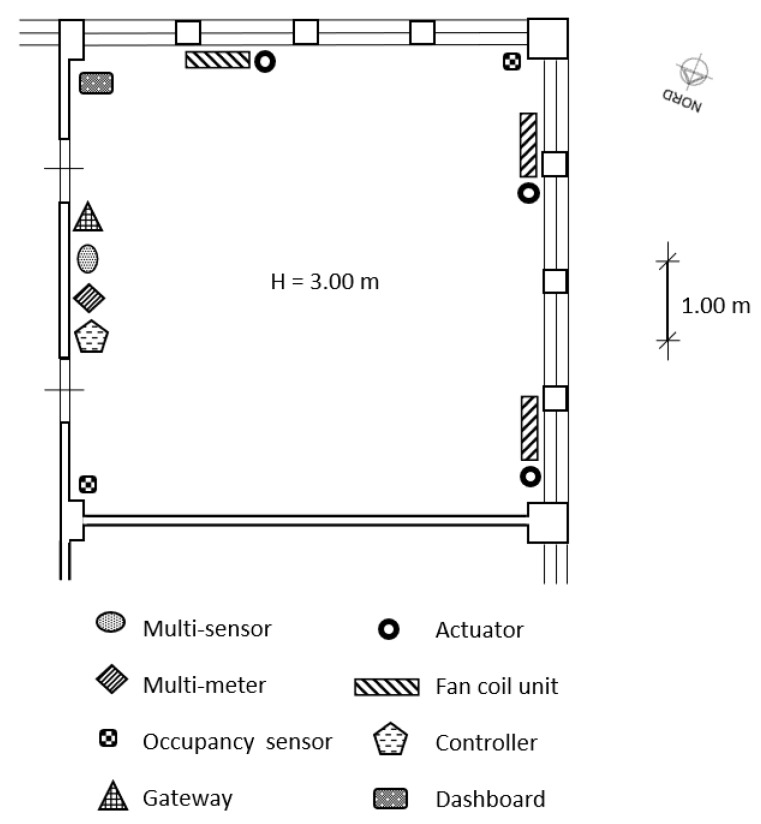
Map of the demo site, with indication on the system main components localization.

**Figure 4:**
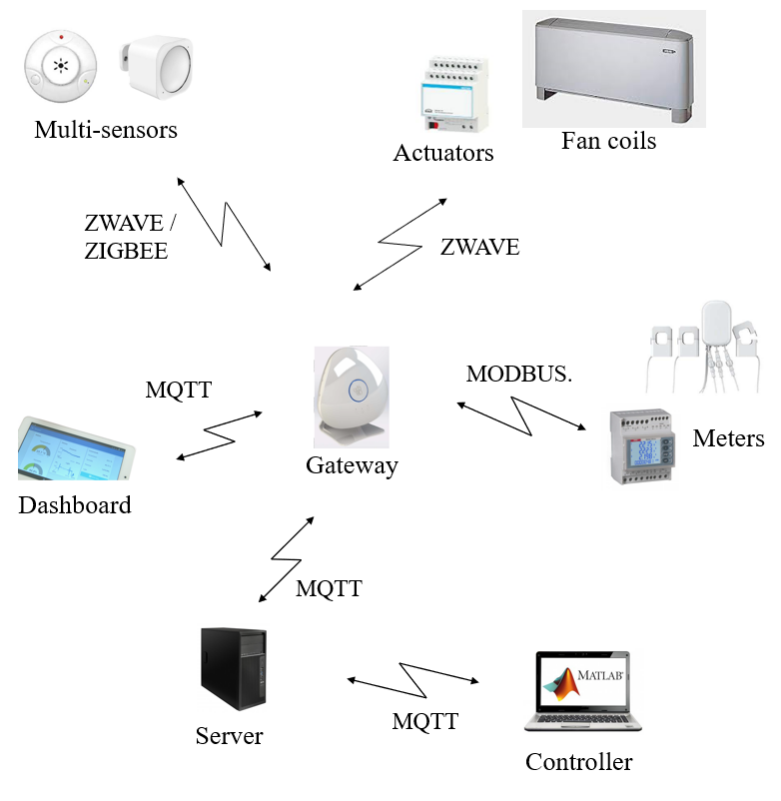
Architecture of the deployed experimental system.

**Figure 5:**
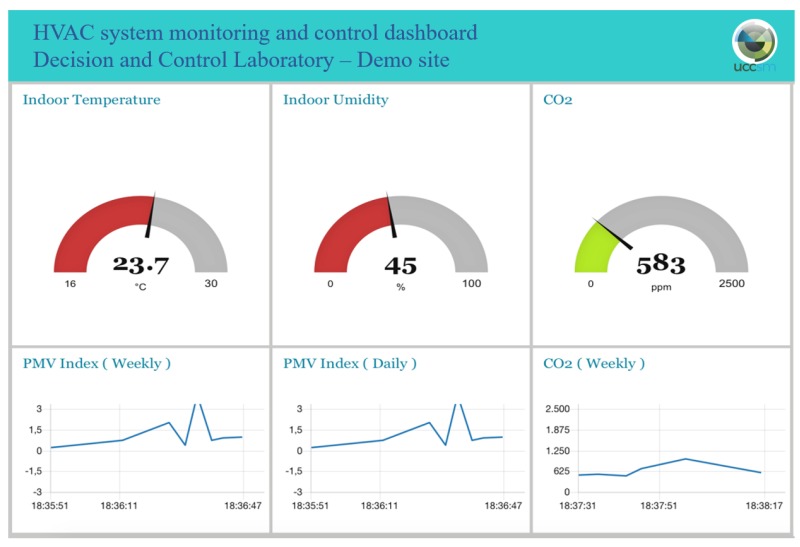
Excerpt of the monitoring and control dashboard prototyped for the demo site.

The proposed IoT based control architecture is implemented on a 36 m${}^{2}$ laboratory located at the third floor of the building and oriented towards North (Figure 3). The laboratory presents three FCUs, whose fan speed can be regulated on-remote. The laboratory is typically occupied by five researchers and doors and windows are often closed; the FCUs provide an indirect and uniform air flow to all the occupants. Figure 3 shows the position, type, and number of installed sensors and actuators. All these devices are Commercial Off-The-Shelf components (COTSs). The system structure follows the framework presented in Section 3. In particular, Figure 4 shows in detail the overall architecture of the experimental system deployed in the above mentioned demo site and described in the following. A multi-protocol gateway, named Beeta Box [33], receives data from all the sensors installed in the room and forwards them to a database server, where data can be accessed by users and applications. The MPC algorithm runs on a control unit that accesses the database server and uses the stored measured data and the weather forecast information as input for the control algorithm. The MPC algorithm is implemented in Matlab on a PC equipped with a 4.0 GHz Intel Core i7 CPU and 16 GB RAM and the weather forecasts are collected from a weather provider through the use of an API [33]. Furthermore, an IP device devoted to end user interface functions is connected to the database server and hosts a dashboard to monitor the state of the environment and set the set-points of the control system (i.e., the comfort temperature, the working hours time window) and the control system mode (i.e., either the standard comfort control based on thermostat, or the MPC algorithm based on the use of PMV, either a manual control to swich on/off the fan coil unit) (Figure 5). The communication standard adopted in the IoT based architecture is the so-called Message Queuing Telemetry Transport (MQTT), which is a message oriented information exchange scheme based on TCP (Transmission Control Protocol) [34]. The quadratic optimization problem Equation (12) is solved using the quadprog Matlab function. The average and maximum computation times are 1 s and 5 s, respectively.

### 5.2. Results Analysis and Discussion

The proposed MPC approach was intensively tested from mid-May 2019 to mid-September 2019, with the HVAC system set to the cooling mode. During this test period, the performance of the presented control algorithm was assessed through an in-depth data acquisition and analysis phase. In the former hot season, the demo site was preliminarily submitted to an ex-ante monitoring phase, aimed at assessing the performance of the previously installed standard thermal comfort control system, which was based on the use of traditional thermostats. This preliminary data acquisition was carried out in the same period of the year (i.e., from mid-May 2018 to mid-September 2018). In particular, the two indicated time-periods proved to be suitable to a meaningful comparison, since both the weather conditions and the average occupancy level of the laboratory were comparable. In the whole test period, the sample time (i.e., Δh) was set to 2 min, whilst the prediction and control horizon were both set to 4 h (i.e., *J* = 120).The occupants’ preferences on thermal comfort were translated into the following requirements on PMV: $P_{ref}\left(j\right)=0$, $P_{min}\left(j\right)=-0.5$, and $P_{max}\left(j\right)=-0.5$, limited to all the time slots *j* corresponding to working hours from 9:00 a.m. to 4:00 p.m. in working days. The working window was also used to set the μ(j) parameter in the Equation (15). Note that the air velocity used in the prediction of PMV was assumed to be constant. In fact, we assumed that, in standard operating conditions, all the doors and windows were closed and all the occupants were sitting at a distance from the fan coil air jets. Considering some parameters, such as the air velocity, affecting the PMV computation constant the estimate of the actual number of occupants was treated as a modelling error in the MPC. The values of model parameters were: η=0.9, $\left|Q_{f}\right|=12\;\frac{Ws}{m}$, τ=3600s, λ(j)=0.5 from 12:00 p.m. to 3:00 p.m. (i.e., to maximize the possible surplus of photo-voltaic production) and λ(j)=1 otherwise, whilst the weighting coefficients in the objective function of Equation (15) are: $w_{1}=1$, $w_{2}=1$, $w_{3}=1$, and $w_{4}=1000$ (i.e., the first three objective function terms are considered equally important, whilst the weight of the fourth term is considerably higher since it relates to a relaxed constraint).

**Figure 6:**
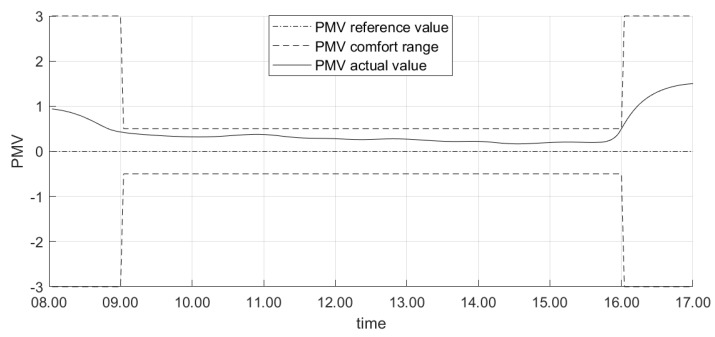
The PMV profile in the demo site in a midweek day in June 2019.

**Figure 7:**
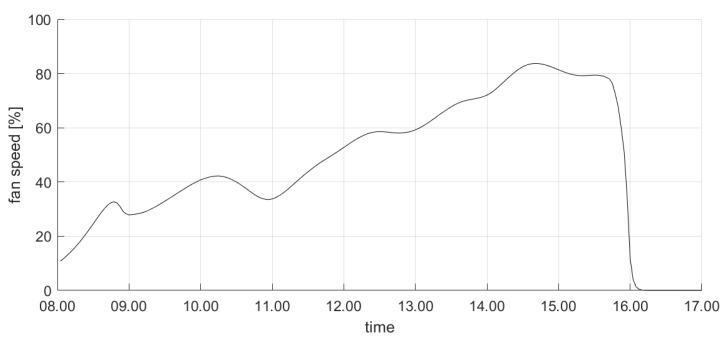
The fan speed profile in the demo site in a midweek day in June 2019.

**Figure 8:**
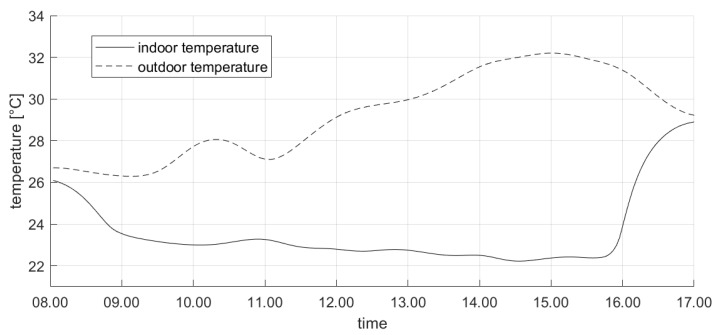
The actual indoor and outdoor temperature profile in a midweek day in June 2019.

For the sake of brevity, we first show the results about the test conducted on a midweek day in June 2019. Figure 6 shows the profile of the controlled variable (i.e., the PMV) during the day: it is apparent that the proposed control system drives the actual PMV value to be limited in the comfort range imposed by the occupants during working hours. Conversely, outside this range the thermal comfort limits are violated. In particular, we remark that the controller activates the actuators (i.e., turns the fans on) earlier than 8:00 a.m. This ensures that the value of PMV enters in the desired zone at the beginning of the planned working window (i.e., 9:00 a.m.). This occurrence can be also noticed in Figure 7, where the evolution of the control variable (i.e., the fan speed) is shown. It is apparent that the fan speed is increased well in advance before the beginning of the working period, in order to achieve the comfort target on time; conversely, the fan speed is reduced roughly at the end of the working period, to ensure satisfying the comfort needs. We also remark that no rapid oscillations in fan speed can be observed in Figure 7, since the limitation in the rate of change is included as a criterion in the objective function in Equation (15). Finally, Figure 8 shows the evolution of the most significant variables that influence the controlled variables. In particular, Figure 8 illustrates the actual outdoor temperature (which impacts the indoor environment thermal model) during the considered day, together with the indoor temperature (which is the primary factor influencing the PMV computation).

We finally report in Table 2 the performance comparison between the proposed MPC algorithm based on PMV and the previously installed thermal comfort control system based on the use of traditional thermostats. In the latter case the thermal comfort was imposed setting the indoor temperature target value equal to 22 °C during the office working hours. First, Table 2 shows in the third column the average daily energy consumed in both scenarios over the whole cooling season. As expected, the MPC approach allowed reducing the energy consumption by approximately 15–20% [10] (specifically, the average daily energy saving was 18.6%). This result is due to the inclusion of the energy saving criterion in the objectives of the cost function to be optimized over the receding horizon in Equation (15). In addition, Table 2 reports in the fourth column the average rate of comfort satisfaction (evaluated as the percentage of working time window in which the PMV is in the comfort range [−0.5, 0.5]) in both scenarios during the office working hours over the whole cooling season. It is apparent that the MPC approach outperforms the programmable thermostat approach. This result is due to the fact that the proposed IoT approach directly uses the PMV as a control variable instead of the temperature; moreover, the comfort satisfaction is timely ensured by predicting the dynamic thermal model of the indoor environment and using this prediction in the optimal control.

## 6. Conclusions

Ensuring the energy efficiency of HVAC systems is one of the main prerogatives of energy managers. The application of automatic techniques can largely reduce the HVAC systems energy consumption without neglecting the necessary comfort requirements. In this paper we present an IoT based architecture for Model Predictive Control (MPC) of HVAC systems that optimizes the indoor thermal comfort and the energy consumption. Differently from the state of the art, we consider the HVAC control system as a whole; that is, on the one hand we provide an IoT based general structure for the implementation of the HVAC automatic control in a real environment, on the other hand we define the MPC optimization problem for the effective control of the HVAC system. The IoT infrastructure is composed by smart sensors and actuators, a gateway, a database server, a control unit, IP dashboards, and APIs that are all connected to the Internet. The measurements and control data are stored and retrieved by/from the field devices, the control unit, and the dashboards to/from the database server; further, thanks to the dashboards, end-users are included in the control loop and can set the comfort temperature and the control system mode. The proposed MPC algorithm is based on a tractable dynamic thermal model of the indoor environment and on a linearized version of the PMV thermal comfort index. In particular, the presented optimization problem provides proper control actions to simultaneously optimize the thermal comfort, the energy efficiency, and the slope variation of the actuation variable, resulting in a flexible quadratic programming problem. As a proof of concept, an experimental system implementing the proposed control architecture is deployed in a real environment inside a campus building at Polytechnic of Bari (Italy). The achieved results show the ease of use of the approach and the effectiveness of the underling control algorithm. In particular, indoor comfort is guaranteed in spite of the presence of multiple disturbances (such as the variation of occupants and the opening of windows and/or doors), and significant energy savings are obtained with respect to standard control approaches based on the use of classical thermostats.

This study is not without limitations. First, the performance assessment of the proposed IoT based control architecture for HVAC systems has not included a cost analysis both in terms of energy and implementation. Future research will be devoted to take such a kind of analysis into consideration, in order to provide the proposed system with a more mature technology/manufacturing readiness level. Second, one can observe that input data such as humidity and occupancy level suffer from estimation uncertainties. Further research will investigate enhancing the model with the prediction of occupancy level based on sensors measurements. Furthermore, the use of robust MPC approaches will be investigated to more effectively cope with uncertainty on model inputs and parameters. Third, the proposed approach has been developed from the standpoint of thermal comfort and energy optimization of a single zone. Further research will also address this issue by extending both the model and the control algorithm to a multi-zone scenario. Finally, distributed/decentralized MPC techniques will be also considered for multi-zone thermal comfort and energy optimization, thus guaranteeing a global thermal and energy efficiency for entire buildings despite the reciprocal influences and coupling between adjacent indoor environments.

## Figures and Tables

**Table 1:** Variables and parameters influencing the PMV.

Variable	Description	Unit
*M*	Energy metabolism	$\frac{W}{m^{2}}$
*W*	Effective mechanical power	$\frac{W}{m^{2}}$
$I_{cl}$	Thermal insulation of clothing	$m^{2}\frac{K}{W}$
$f_{cl}$	Air coefficient of clothing	dimensionless
*T*	Indoor air temperature	°C
$T_{mr}$	Average radiant temperature	°C
$v_{ar}$	Relative air speed	$\frac{m}{s}$
$P_{a}$	Partial pressure of water vapor in the air	Pa
$h_{c}$	Coefficient of heat exchange by convection	$\frac{W}{K\;m^{2}}$
$T_{cl}$	Surface temperature of clothing	°C

**Table 2:** Comparison of thermal control systems tested in the demo site.

Type of Control Systems	Period of Analysis	Average Daily Energy Consumption [kWh]	Comfort Satisfaction during Working Time [%]
Programmable thermostats	from mid-May 2018 to mid-September 2018	16.74	75.1
MPC	from mid-May 2019 to mid-September 2019	13.63	95.4
